# Supplementary material for: Anomalous diffusion analysis of semantic evolution in major Indo-European languages
Source: PLoS One. 2024 Mar 26;19(3):e0298650. doi: 10.1371/journal.pone.0298650 (PMC10965085; doi:10.1371/journal.pone.0298650)
Supplement: S1 File — We present and explain in this file all the auxiliary results and arguments that are related to this paper. (PDF) [file pone.0298650.s001.pdf]

Anomalous diffusion analysis of semantic evolution in major Indo-European languages

Supporting information

Bogdán Asztalos
Dept. of Biological Physics, Eötvös University, H-1117 Budapest, Pázmány P. stny. 1/A, Hungary

Gergely Palla
Health Services Management Training Centre, Semmelweis University, H-1125 Budapest, Kútvölgyi út 2, Hungary
Dept. of Biological Physics, Eötvös University, H-1117 Budapest, Pázmány P. stny. 1/A, Hungary

Dániel Czégel
Parmenides Center for the Conceptual Foundations of Science, Hindenburgstr. 15, 82343 Pöcking, Germany
Institute of Evolution, Centre for Ecological Research, H-1121 Budapest, Hungary

S1 Dual space concept of the Skip-gram model

Skip-gram model is a word embedding method using a two-layer neural network. During training, two D-dimensional representations are constructed for each word, one in the hidden layer, and the other one in the output layer. Since these two representations are related only through their scalar product in the estimation of conditional co-occurrence probability, as seen in Results, they can be considered to be elements of a vector space and its dual space. This perspective provides a couple of consequences that were used in our argument in the paper. Here, we formalize this dual space perspective and introduce some of its useful interpretations.

Let S be the set of words i.e. the *vocabulary* and V be a finite D-dimensional vector space, the *word vector space*. Constructing a word embedding means assigning a $v_i \in V$ vector to any word i as its representation, or in mathematical language, defining a $v : S \to V$ map. In practice, this map is stored in the hidden layer of the training neural network.

For a given language with the vocabulary S above, a bivariate function $p : S \times S \to [0, 1]$ can be defined over ordered pairs of words, with a value equal to the probability that the first word occurs in the context of the second one. (E.g. $p(\text{cat}|\text{dog})$ gives the probability of finding the word *cat* in the context of the word *dog*.) Using this, we can define another function as

$$\begin{aligned} f_j &: v\,[S] \to \mathbb{R} \\ f_j\,(v_i) &:= \ln p\,(\,j\,|\,i\,) \end{aligned} \tag{S1}$$

where $v\,[S] \subset V$ is the image of S under the function v, and j is a given word from S.

If the number of words is significantly larger than D, then we can presume that the generated subspace of $v[S]$ is the whole V word-vector space. Let us choose D independent word-vectors $\{v_{i_1}, \ldots, v_{i_D}\}$ and since they form a basis on the generated subspace, all the word-vectors can be expressed as their linear combination:

$$v_j = \sum_{k=1}^{D} \lambda_k^j \cdot v_{i_k} \tag{S2}$$

with the suitable λ_k^j coefficients. If f_j is linear, i.e. if

$$f_j\left(v_l\right)=\sum_{k=1}^{D}\lambda_k^l\cdot f_j\left(v_{i_k}\right)\quad\forall l\in S, \tag{S3}$$

then it can be uniquely extended to the whole V vector space as a linear functional, so it is an element of its dual space V^*. Because of the finite dimensional Riesz representation theorem, f_j can be represented by another vector $w_j \in V$ fulfilling the identity

$$f_j\left(v_i\right)=v_i\cdot w_j\qquad\forall i\in S. \tag{S4}$$

If identity (S3) holds for all word j, then besides the word-vector map v, there exists another map $w : S \to V$ which can be viewed as the representation of words as contexts. Using the definition of f_j, the identity (S4) is equivalent to the log-probability estimation of the Skip-gram model seen in Results, so the assumption the Skip-gram model actually makes is the linearity of the log-probability of the conditional co-occurring in its second variable.

If our presumption does not hold, and $v[S]$ generates a d-dimensional subspace of V, where $d < D$, then only an d-dimensional dual space can be uniquely defined, but it can be extended to D dimensions. Though this extension is arbitrary, the formalism above can be done.

It is important to note that though the w_i context vectors are also elements of the vector space V, their role emerges only when they are multiplied with word vectors (as a word from S also can only be in the role 'context' if there is another word whom it can be close to). Hence, context vectors cannot be viewed as irrespective vectors despite the fact that in practice they are stored in the output layer of the training neural network, similarly to the word vectors in the hidden layer. They are just the representations of the functional elements of the dual space V^*.

Also, however, this long, axiomatic-like deduction leads to the well-known formalism of the Skip-gram model, there is an important difference to the usual model definition. The Skip-gram model uses context-vectors as a second representation of words, but in fact, their role is more functional-like than vector-like. They are necessary not for representing words in a second way, but instead for assigning probabilities to the existing word representations.

S2 Arbitrary sample size

According to our experience, the time-labeled data downloaded from the Google Ngram database shows a steady increase in the number of yearly word occurrences time probably due to the phenomenon that newer text sources are more available, have less probability to disappear or become obsolete and so could digitalized and processed by Google more easily. Nevertheless, this sampling bias has an important consequence on our data processing, because the output of the Word2vec implementation used by us is very sensitive to the training data size. To examine its effects, we subsampled the co-occurrence statistics of the words and constructed word embeddings using the subsampled input data. (More precisely, we performed the steps shown in Figs 2f-h, i.e., we constructed $M = 80$ different embeddings, aligned them, and took their average, as described in Methods, but using different N_c values.) In Fig S1 we plotted some of the geometric properties of these "subsampled embeddings" in the function of N_c.

This figure shows that using more than about $2 \cdot 10^6$ training wordpairs, the size of the word cloud grows monotonically, but since the uncertainty of word positions remains reasonably low, the reliability of these averaged word positions remains valid. Moreover, Fig S1 also reveals that the position uncertainty with respect to the radius and the AOV of the word cloud from the origin converge to constant values, so using more and more input data, its structure and stored information held by a single embedding remains about the same, only its size expands. This can be interpreted as the size of the input data affects only the distance measure with a simple linear multiplicative factor. Hence, to conserve the comparability of consecutive embeddings, one needs to fix the distances which can be done most easily by applying the same number of samples as input. This number needs to be chosen arbitrarily and was $N_c = 10^7$ in our pipeline, because – as can be seen in Fig S1 – the cloud structure is already reached at this sample size, and we did not want it to be too large in order to shorten the runtime.

S3 Semantic drift of stopwords

During the data processing phase of our method, we filtered out stopwords from the linguistic data before any statistical analysis as mentioned in the Methods. This is a usual step in the field of NLP to avoid undesired bias caused by frequent grammatical words that are insignificant from the point of view of semantics as their purpose is more functionary than representative of meaning. At the same time, one can ask if they have any role in the phenomenon studied by us, and if they contribute in any way to the semantic drift. To verify this, we rerun the processing pipeline on the English data

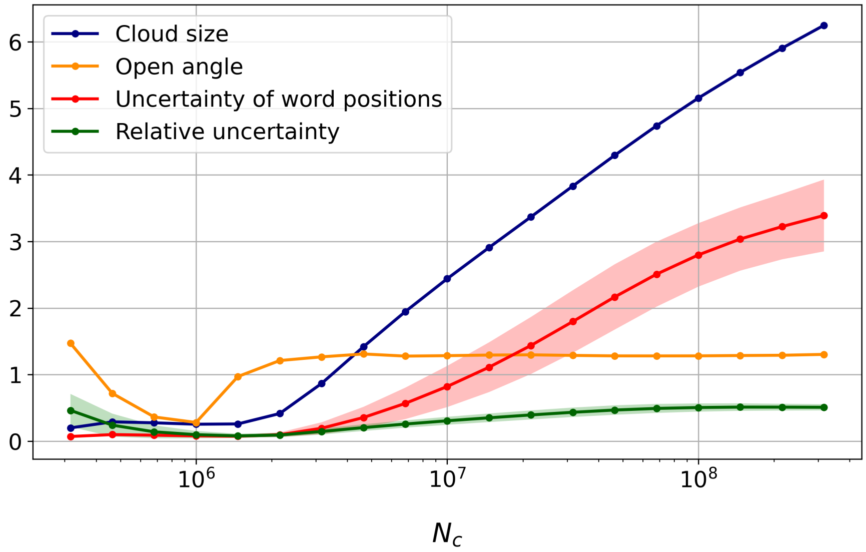

S1 Fig. Some basic geometric properties of the embedded word cloud in the function of the number of co-occurring word pairs N_c we used as input for training. *Cloud size:* The quantity defined in Eq. (11) in the paper which characterises the spatial extent of the set of the embedded words. *Open angle:* The open angle of the smallest D-dimensional hyper-cone whose vertex is fixed to the origin and contains all the embedded word positions. If the origin was inside the cloud, the angle would be obtuse, but this was never the case. *Uncertainty of word positions:* The spatial deviation of the M embedded vectors corresponding to an individual word after the alignment. This can be considered as the spatial uncertainty of its position. (The solid line shows the mean value over the words, the shaded area the standard deviation.) *Relative uncertainty:* The uncertainty of an individual word divided by its distance from the center of the word cloud (i.e. the radius). (The solid line shows the mean value over the words, the shaded area the standard deviation.)

except for the stopword filtering step (Fig 2b) and plotted the average and some individual squared displacement $(\Delta x)^2$ in the function of time in Fig S2.

The results show that the subdiffusive character of semantic changes has remained, and the overall shape of the time-dependence is also about the same in Fig S2 as earlier in Fig 3a. We can also notice that the curve representing the average of stopwords is almost exactly the same (apart from a constant factor) as the one corresponding to the total average, and the individual words also follow this shape relatively well. In the light of the fact that semantic displacements and trajectories cannot be interpreted individually only collectively as discussed in the Methods this is understandable, because since stopwords have no semantic meaning they adapt to the embedded environment, and adjust to the subdiffusion shown by the other words. At the same time, one must be careful in drawing conclusions about the semantic behavior of stopwords, since they have no classic semantic meaning, so talking about the change of it is very vague and equivocal. In any case, it can be stated that the presence of stopwords does not change qualitatively the results discussed in the paper.

S4 Randomized trajectories for all five languages

In the paper, we discussed the displacement over time of words compared to their randomized versions and found a subdiffusive behavior, which can be observed through both the ensemble average and the mean of single trajectory anomalous diffusion exponents $\langle \alpha \rangle$ and $\bar{\alpha}$. These results were visualized in Results where we plotted the squared distance–time function and the distribution of exponents of English words. Since the qualitative characters of these figures were similar for the cases of all five studied languages, we did not insert them into the paper, but they can be seen here in figures S3 and S4.

S5 The effect of sliding time window size

As explained in the paper, temporal data was grouped into 10-year-long periods which slid only by years, so 90% of neighboring time periods data had overlapped with each other. The purpose of this was to smoothen the measured

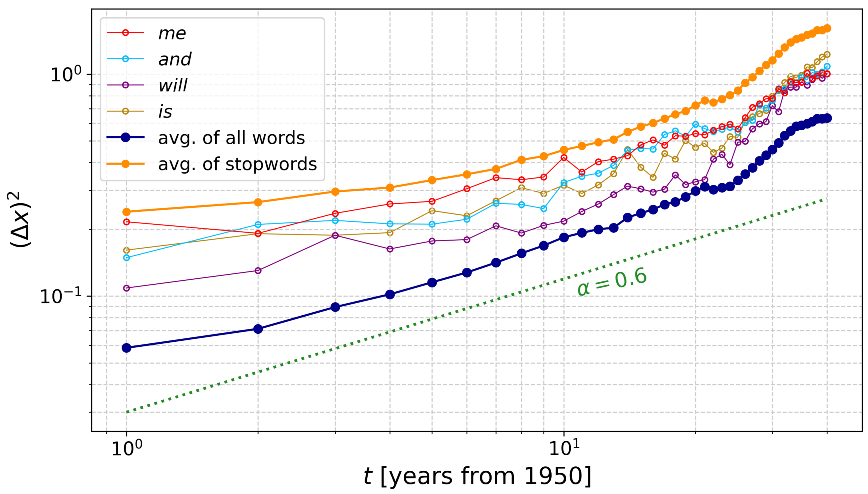

S2 Fig. Squared displacements in the case where stopwords was not thrown away. Squared displacement $(\Delta x)^2$ of the average squared displacement of all words over time, along with the average of stopwords, and some selected stopwords. It can be seen that although the slope of the curve has increased, the subdiffusive character of the semantic drift has remained.

quantities in time, because the sharper the resolution was, the more noisy the measured distances were, and this made the determination of the anomalous diffusion exponent more difficult. This effect appeared to be the strongest in the case of the English data.

In order to study how the arbitrary window size affects the subdiffusive behavior we see, we applied some different values of the window size and trained new word embeddings with the newly grouped linguistic data. The result was that though the actually measured anomalous diffusion exponent value changed, the result still remained significantly under $\alpha = 1$, thus, the subdiffusive behavior seems to be independent from the temporal grouping. In the left panel of Fig S5 we plotted the measured $\langle \alpha \rangle$ ensemble average anomalous diffusion exponent as the function of the sliding window size across the five studied languages, and the presumed trend can be observed: longer sliding windows smoothen the noisy motion and hence increase the exponent, in all five languages, however, this increase slows down with longer window sizes and the exponents approximately converge to a value $\langle \alpha \rangle \approx 0.4$ to 0.5, corresponding to sliding window size 10.

S6 Results with shuffled time labels

As we explained in the paper, temporal alignment of consecutive embeddings is inevitable due to the abstract sense of the embedding space and the stochasticity of the embedding algorithm, and this alignment may add temporal correlation between different time states. This suggests that starting even from temporally independent data, one will be able to find some (unreal) temporal trend caused by this necessary technical step of the processing pipeline. To make sure that the findings we discussed in the paper are not the results of such methodological effect, we performed the whole processing and embedding procedure on a temporally uncorrelated dataset.

The generation of a temporally uncorrelated linguistic dataset from scratch would be challenging since many statistical features must match the original data for the embedding algorithm to produce a meaningful result. Instead, we kept the individual yearly co-occurrence statistics and permutated their time labels. In this shuffled time series of co-occurrence data a real time state corresponds to each year, but their temporal correlation got eliminated due to the random permutation. Without temporal alignment consecutive word positions would be independent of each other, so we would expect the squared distance $\left|(\Delta x)^2(t)\right|$ to show no tendency in time, and the anomalous diffusion to be zero.

The observed displacement trends obtained this way can be seen in the right column of Fig S6 while in the left columns, the real displacement trends are shown as a comparison. The fitted $\langle \alpha \rangle$ ensemble average exponents are plotted in the right panel of Fig S5.

It can be established that the value of the $\langle \alpha \rangle$ exponent is around 0.2 to 0.3 for each language and window size, only in the cases of English and Spanish can a slight increase be observed (but the exponent of the real English data also drops down to such a low value, so that is also expected for the shuffled case). Nevertheless, using longer window sizes (where the signal-to-noise ratio is lower) and all shuffled exponents are significantly smaller than the real ones. Also, if

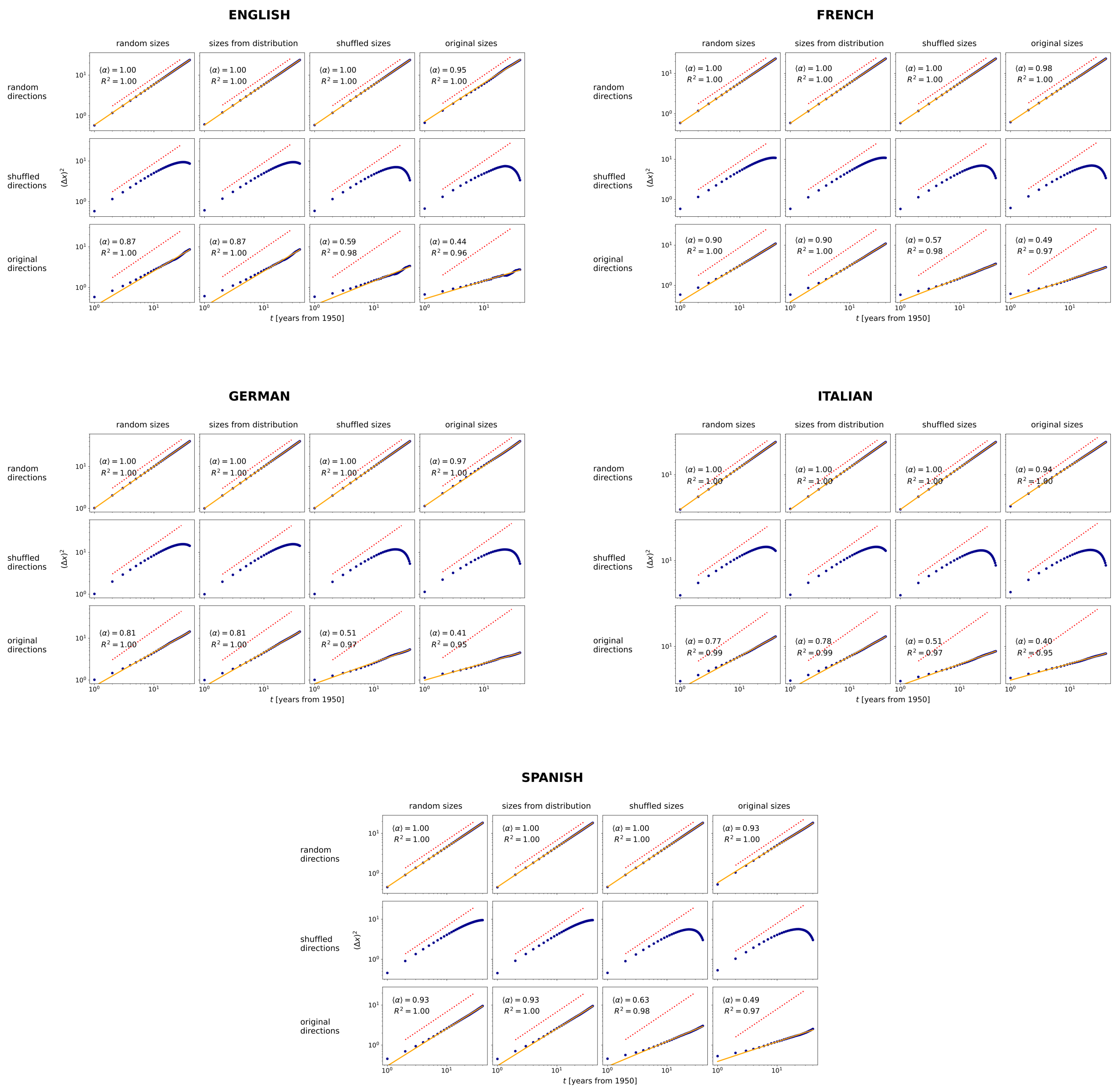

S3 Fig. Reproduction of Fig 2c for all five languages. I.e. ensemble-average squared displacement $\langle(\Delta x)^2\rangle$ of words under various combinations of trajectory randomization methods.

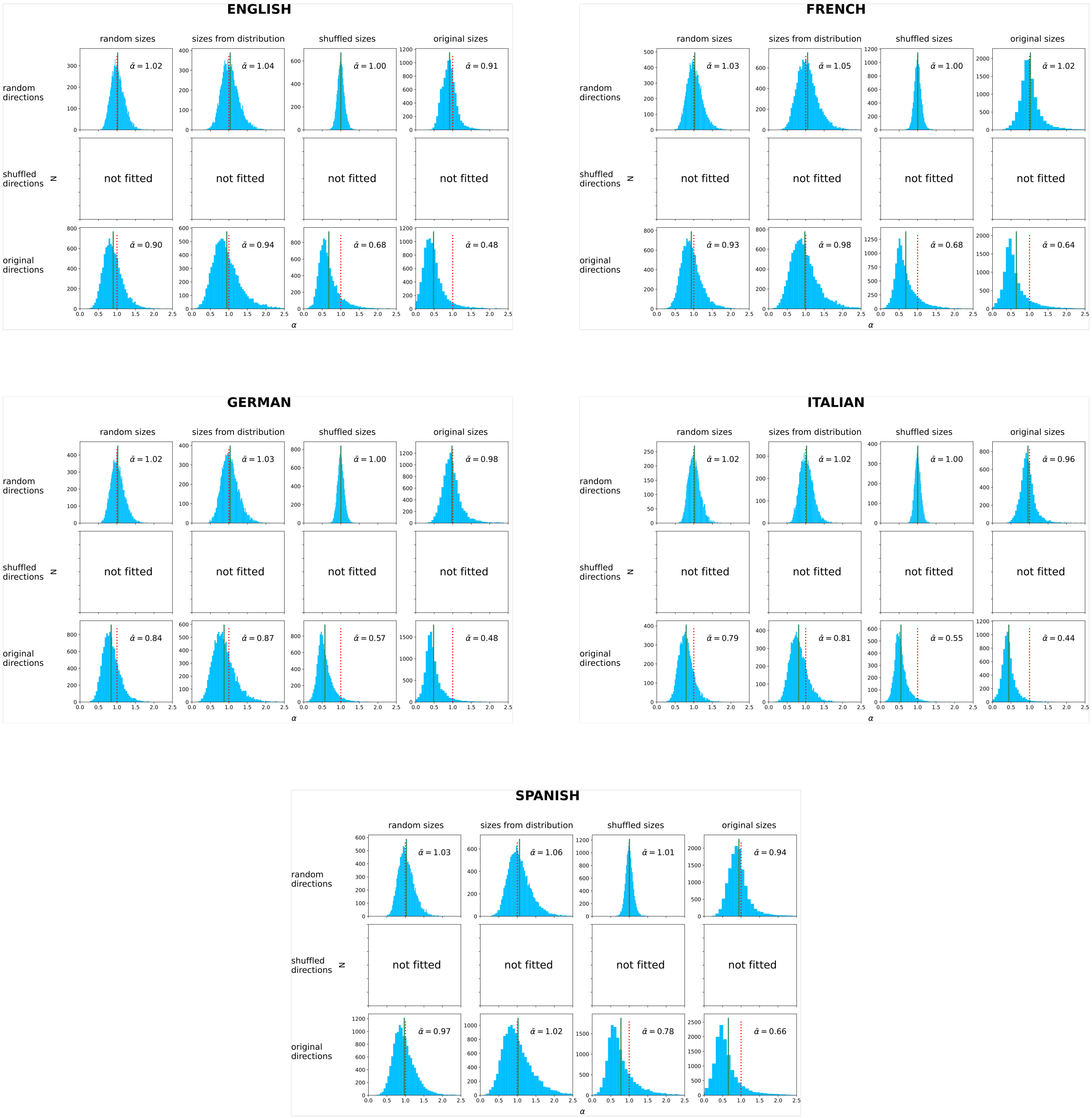

S4 Fig. Reproduction of Fig 2d for all five languages. I.e. distribution of anomalous diffusion exponents α fitted to the trajectory of each word separately, under various combinations of trajectory randomization methods.

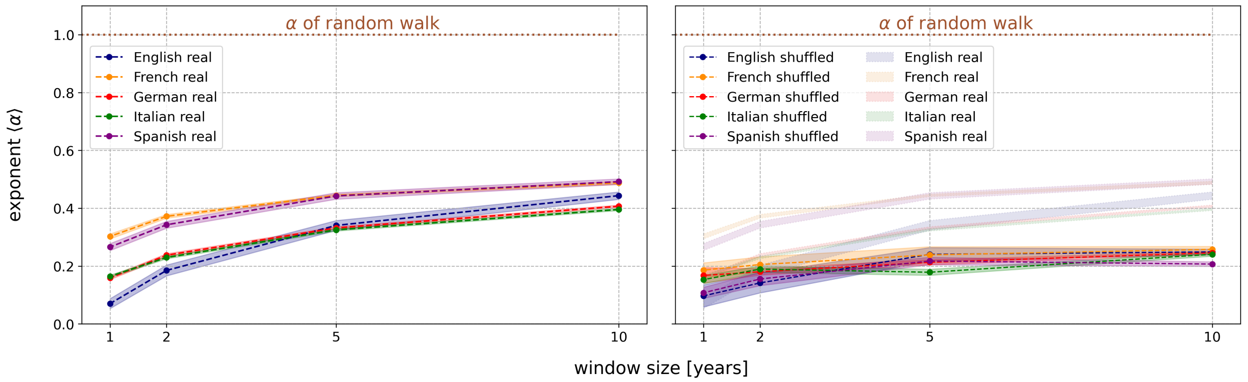

S5 Fig. The measured $\langle\alpha\rangle$ values and their uncertainty in for various temporal sliding window sizes. In the left panel results obtained from real data are shown, while in the right panel results from data with shuffled time-labelled.

we look at the distancing data in Fig S6, we can see the shuffled data has much higher fluctuation than the real one. While the real data shows very smooth distancing trends, the bouncing data points in the shuffled case make the whole climbing trend uncertain. This is received also if we measure the R^2 value of the exponent fitting (shown in the upper row of Fig S7), where all cases of real data have almost exactly $R^2 \approx 1$ (except for English), while the shuffled cases showed much lower values, even for longer window sizes. We also measured the relative fluctuation of the $(\Delta x)^2(t)$ data points (shown in the bottom row of Fig S7), and it also suggests that while real data fluctuates almost nothing (again, except for English), significantly higher values correspond to the shuffled cases.

We also compared these results with the case when the time labels of diffusing ($\alpha = 1$) random walk (showed on Fig 3b) are reshuffled before the alignment. As can be seen in Fig S8, a non-zero anomalous diffusion exponent of similar magnitude can be measured indicating that the alignment processing step creates a stable background with an $\langle\alpha\rangle$ exponents value $\sim 0.2 - 0.3$. The observed phenomenon characterised by the $\langle\alpha\rangle \approx 0.4 - 0.5$ value is unequivocally distinguished from it suggesting that the observed subdiffusive phenomenon origins from real language characteristics.

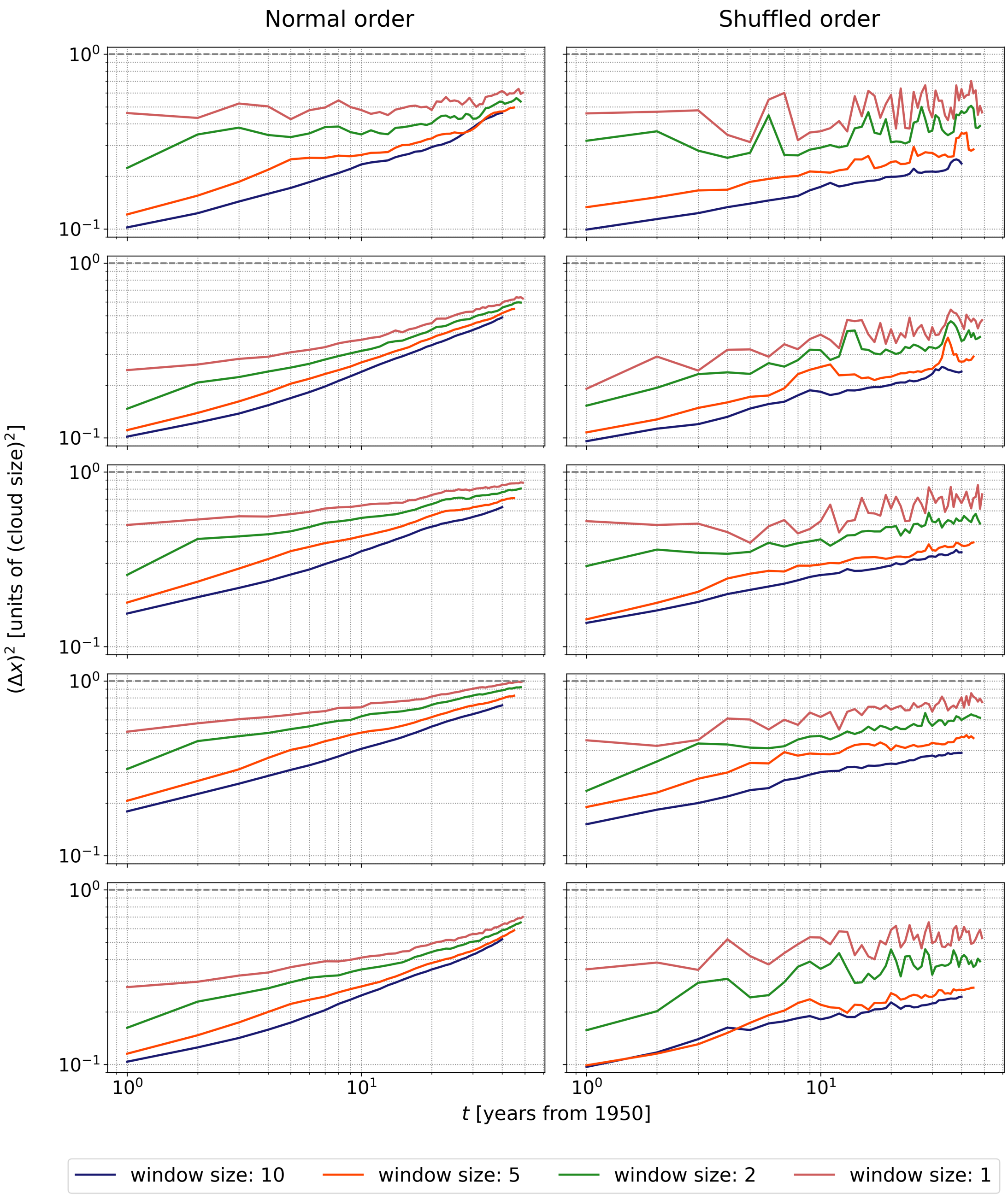

S6 Fig. Squared displacements in the case of shuffled time labes versus the original data. The ensemble-average squared displacement $\langle(\Delta x)^2\rangle$ of words in the cases of different languages (rows) applying different sliding windows (colors). The results shown in the left column are obtained from the real time-dependent data series, while in the right column, the results are obtained from the pseudo-diachronic data series which was created by shuffling the time labels in the raw data. (Since the size of word clouds differed from each other in separate time series, we normalized the distance measure, and defined the unit as the cloud size.)

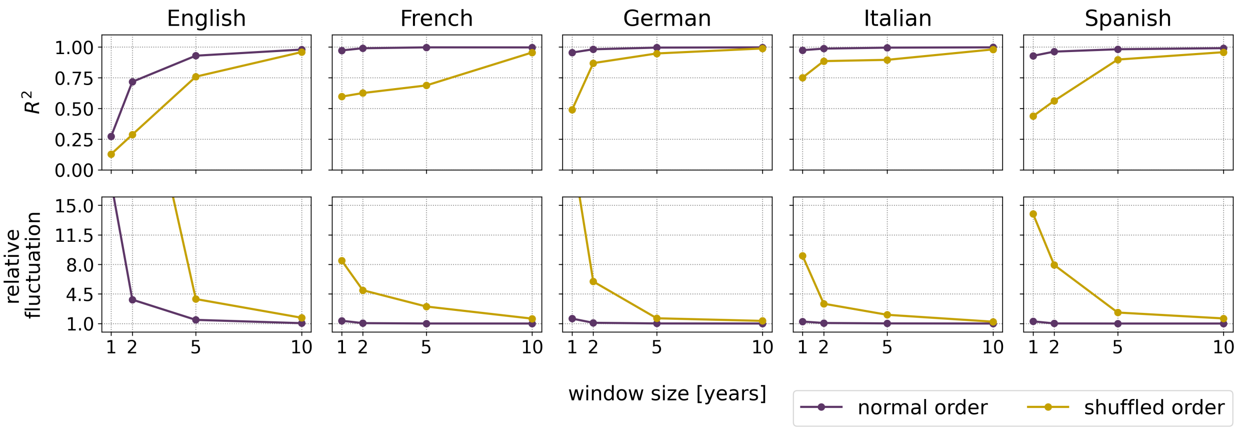

S7 Fig. Fluctuations of the squared displacement $(\Delta x)^2(t)$. *Upper row:* The coefficient of determination R^2 of the fitted power function on the linguistic data shown in Fig S6 which measures how well the subdiffusion model describes the observed data. *Bottom row:* The relative fluctuation of the linguistic data shown in Fig S6 calculated by $\left(\sum_t \left|(\Delta x)^2(t+1) - (\Delta x)^2(t)\right|\right) / \left(\sum_t (\Delta x)^2(t+1) - (\Delta x)^2(t)\right)$ which measures how strong the fluctuation is in the observed data.

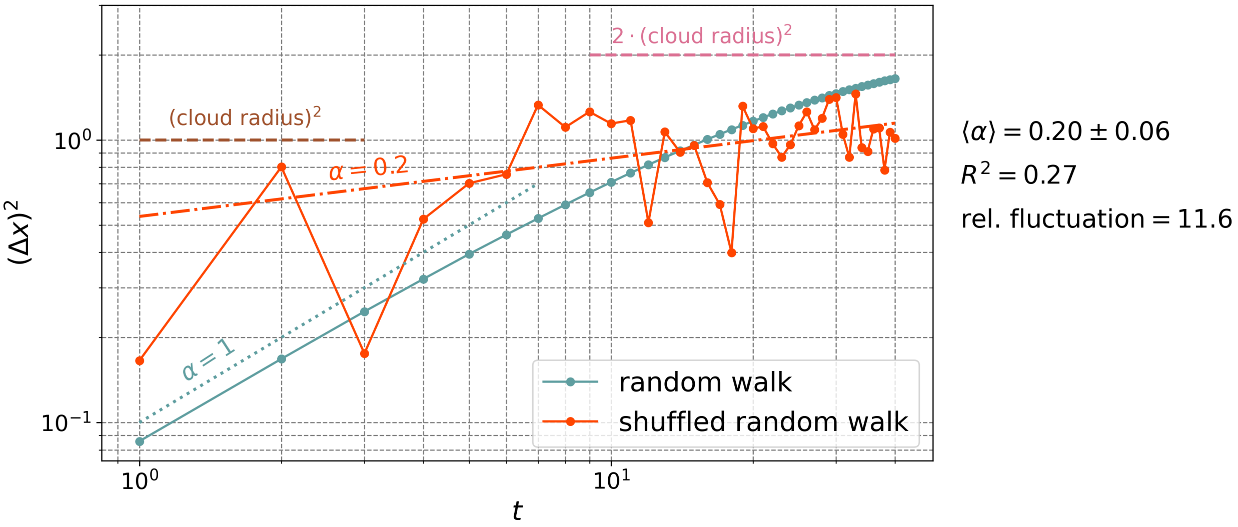

S8 Fig. Squared displacements in the case of diffusing random walk with and without shuffled time labels. The ensemble-average squared displacement $\langle(\Delta x)^2\rangle$ of words in the case of the random walk from Fig 3 (light blue), and in the case of when the time label shuffling is applied on it. The fitting results of the latter case can be seen on the right side.
