# Supplementary material for: Anomalous diffusion analysis of semantic evolution in major Indo-European languages
Source: PLoS One. 2024 Mar 26;19(3):e0298650. doi: 10.1371/journal.pone.0298650 (PMC10965085; doi:10.1371/journal.pone.0298650)
Supplement: Attachment — Submitted filename: response_letter.pdf [file pone.0298650.s002.pdf]

Beyond Center for Fundamental Concepts in Science,
Arizona State University

Dániel Czégel
Beyond Center for Fundamental Concepts in Science
Arizona State University
Tempe, AZ 85287, USA

October 19, 2023

Haroldo V. Ribeiro
Academic Editor, PLOS ONE

Dear Haroldo V. Ribeiro,

thank you very much for your e-mail message of 8th of August 2023, related to the review of our manuscript with Submission ID PONE-D-23-19326 entitled "*Subdiffusive semantic evolution in major Indo-European languages*" by Bogdán Asztalos, Gergely Palla and Dániel Czégel including two mainly positive reviews, suggesting the revision of our paper. Although the referees acknowledged the merits of our research, they have also raised fair criticism regarding certain aspects of our manuscript.

In addition to minor comments and adjustments, Reviewer 1 mainly raised conceptual and interpretive questions, while Reviewer 2 asked us to perform further analysis to support the main claims of the paper. Consequently, the main changes in our resubmitted manuscript are the following:

1. We have added more new sections to the Supporting information in which we place our argument in favour of the subdiffusion on a more stable basis.
2. We have explained the arbitrary methodological choices in more detail.
3. We have expanded the discussion trying to emphasise the relevance of subdiffusion, the conclusions that can be drawn from it, and highlighting the analogies to natural sciences more explicitly.
4. During revision we noticed that the code calculating the standard deviance for the exponents in S5 Fig (Figure S3 in the previous version) unfortunately took the square root of the variance twice, resulting larger deviance values compared to the true value. This is now corrected.
5. Adapting to the journal requirements, we have changed the numbering

and the caption format of the figures in the Supporting information, added these captions to the end of the main paper and removed the founders from the Acknowledgments.

We thank all Referees for their outstanding work and the very valuable comments, making the revised version of the paper significantly better, which we hope is now suitable for publication. Our point-by-point response to the editorial board comments and the remarks by the Referees (reproduced in italics) are given below.

Response to Reviewer 1:

Minor

1)The English must be revised. Some typos (lenght, patters, pyton, psycholiguistic) and other minor grammar issues exist.

We have run a language checker on the entire manuscript to correct all the typos and minor grammar issues.

2)Apparently, there needs to be an explicit explanation for why considering data from 1950.

We focused on years after 1950 because taking earlier years into account would have decreased drastically the size of the vocabulary. We have added a few sentences to explain this to the section Temporal grouping in Methods.

Major (i.e., suggestions)

1) The authors present the ensemble-average anomalous diffusion exponent and the average anomalous diffusion exponent. Based on that parameters and in the randomization tests, the authors could mention/investigate the ergodic properties of the systems. The ergodicity concept may shed new light on the author's main arguments and provide a technical way to analyze their data.

Thank you for the suggestion. We have been considering it but in the end decided that robust claims about non-ergodicity would need both significantly more theory and also more analyses about the role of noise (the trajectories are noisier than what typically is available in physical and biological systems). Furthermore, since this paper should address an interdisciplinary audience and mainly raise a question (why subdiffusion?), we suggest that the investigation of non-ergodic properties could be the topic of a follow-up study.

2)The discussion section could better explain or explore the distinction between a usual random walk and the subdiffusion. One of the main results is that the meaning of the words does not change randomly (like usual diffusion) but is somehow correlated with its previous meaning. Such a result is a clear example of memory in diffusive systems. For a particle, we borrow the term "memory," but in the present work, the temporal correlations may be related to the memory (cultural memory). In other words (not a pun), people change the use of the words, not randomly, but considering more or less what they remember of the previous meaning.

This is also a very good point. One of the main issues is that autocorrelation of word trajectories is most likely a result of both collective phenomena and individual memory. For example, in the revised manuscript, we analyze the meaning change of stopwords and find that their average anomalous diffusion exponent is not significantly different than that of all words. This indicates that subdiffusion is primarily a collective phenomenon. As with the previous recommendation, this is a very important topic that deserves an in-depth analysis and is outside the scope of the current paper.

In order to clarify the scope of the current paper, we have expanded the last few paragraphs of Discussion to emphasise the relevance of subdiffusion and the conclusions that can be drawn from it.

3)Moreover, in the works of subdiffusion in biological systems, one of the causes of diffusion is the interactions in the systems (crowding, disorder, geometrical constraints, encounters of particles), and language exists for and because of interactions. The present work has an excellent opportunity to explore such a meaning of memory in anomalous diffusion, which sometimes is impossible to discuss deeply when one considers particles just colliding according to the laws of physics. In summary, the results could bring some insights into interpreting the memory concept (in models of anomalous diffusion).

This was also our vision when we started to work on this subject, and we still hope our work can contribute to the evolution of how people view and interpret memory. However, we do not intend to go directly into this reinterpretation process in detail in this paper, instead just to present our results about subdiffusion that can be used in potential future work. In order to summarize the conclusion about the connection between cognitive memory and the dynamical memory concept in a more unequivocal way, we have rewritten the last paragraph of Discussion highlighting the analogies

to natural sciences more explicitly.

Response to Reviewer 2:

** Main points:*

1) The plots showing evidence of subdiffusion in Fig. 3a show oscillating trajectories that, at best, show an average subdiffusive behaviour for 1 decade (from 2y to 20y). Even the averaged results for different languages in Fig. 3b show a systematic increase in the slope with time. This raises the question of the extent into which subdiffusion is a good description of the observations and, if so, what are the time scales in which it applies? The current evidence in support of the general applicability of a subdiffusive model and Eq. (1) is very weak and the conclusions would need to better reflect these limitations.

The individual trajectories corresponding to concrete words are very noisy indeed, but our scope was to describe their collective behavior through the ensemble average and the mean anomalous diffusion exponents. The quantitative analysis of the separate trajectories would not provide the right kind of information, because they can be viewed only in relation to the others. To make this clearer, we added a new paragraph to the subsection Mean versus ensemble average anomalous diffusion exponent in Methods, where we explain why collective phenomena should be studied instead of individual trajectories. (The plotted trajectories in Fig 3a serve only visualization purposes: they illustrate what the blue curve is the average of, and to show that we can find certain words that change faster than others.)

To quantify how good model the subdiffusion is, we have calculated the R^2 for each time dependence fit where it made sense (see Fig 3c, S3 Fig, and S7 Fig) and in the cases based on real data were very close to 1. Although there are indeed changes in the slope on the studied time scale these do not significantly degrade the subdiffusive model. Collecting data from longer time intervals may give more accurate results, but it cannot be increased in magnitudes even with an adequate amount of data, because the displacement cannot be arbitrarily large (see the answer for point 4) and will converge to $2 \cdot (\text{cloud radius})^2$ as the random walk curve does in Fig 3b. The anomalous diffusion exponent needs to be measured while this convergence is negligible i.e., just for a few decades. To study this phenomenon on a longer time scale, one must use a different methodology.

Related questions to be clarified include:

1a) Why are the authors focusing only on the years from 1950? The co-

occurence is built based on windows of size 2 only, so that information on the Google n-gram corpus should allow (e.g., taking $n = 5$ in the n-gram data) studies going further into the past as there is abundant data up to 1800. This would allow one to clarify whether the scaling in (1) holds with a similar α through longer time scales (the essence of an anomalous diffusion approach). At least a clarification on this point would be important.

We focused on years after 1950 because taking earlier years into account would have decreased drastically the size of the vocabulary. We have added a few sentences to explain this to the section Temporal grouping in Methods.

1b) The effect of the temporal sliding window in these plots is not clear. The size goes up to 10y and is thus comparable to the range in which a straight line is seen in the figures. Figure S3 shows that the effect of using the window changes significantly the value of the average exponent α, strongly suggesting that the straight line fit underlying the computation of individual αs is not a good model for the data.

The exact value of the measured anomalous diffusion exponent depends on the size of the sliding window, but the subdiffusive behavior appears in the cases of shorter window sizes too, where it is not comparable to the range of the straight line (see the left column of S6 Fig). The main result of our paper is intended to be that the semantic evolution is subdiffusive, but since we had to make a lot of arbitrary parameter choices during the steps of the applied methodology, it is not surprising that the precise value of the measured exponent depends on these parameters. However, we argue throughout most of the Supporting information that the subdiffusive behavior itself is a feature of the language, not the pipeline.

2) While the randomization provided are important, I recommend the authors to consider randomizations and data analysis performed at the original textual data so that the effect of the whole pipeline can be better evaluated (also in view of point 1. above). In particular, two simple tests of the results would be to reproduce Fig. 3a for:

2a) Stop words, i.e., maintaining some of the filtering words in step Fig. 2b and evaluate what would be the results for these words. It'd help to better evaluate whether the observed displacement can be indeed associated to semantic drift.

2b) Shuffling the years of each of the corpora, i.e., randomly attributing dates to each of the corpus. By simply changing the years associated to each

corpus and reproducing all steps of the pipeline one should then obtain a good null model to compare the results against.

We have examined suggestions 2a) and 2b) and have included their result into the Supporting information (see sections S3, S6, and figures S2, and S5-S7). We have found no such result that suggests that we should dismiss the subdiffusive description of semantic evolution, and still think that Eq (1) is a good model for the observed data.

** Minor points:*

3) Line 52. I found it puzzling that Ref. [23] is cite here in support of Zipf's law, as it claims that Zipf's law is not valid for words.

The sentence in line 52 states that Zipf's law causes a systematic bias due to the unequal distribution that can be found in language. Ref. [23] reformulates the statement of the original Zipf's law (formulated in Ref. [22]) from words to phrases. It does not invalidate the existence of Zipf's law nor the bias caused by it, just gives a more accurate form of it.

4) Methods section: is there any upper or lower bound on $|\Delta x|$ or on the vectors $|v|$? This would be important to evaluate limits on the subdiffusive behaviour.

In theory, an individual word can go arbitrarily far from its initial position, but the size of the whole word cloud, defined in Eq (11), is fixed by the constant sample size N_c. This means that the number of words that significantly moves away from the cloud is strictly limited, and our experience is also that the length of individual displacement vectors are typically not much larger than a few times of the cloud size, and their mean square would converge to $\sqrt{2}$-times the cloud size after long time.

5) Lines 189-191: the current wording suggest that Fig. 2f shows that subsample is needed to correct the sample size bias. In reality this is not the case and it remains unclear why equating the sample size is needed and whether $N_c = 10^7$ provides an appropriate choice.

We have added a new section to the Supporting information where we examine the effects of the arbitrary sample size and show that the sample size bias causes problems that should be corrected. We have also added another sentence after the mentioned part in the paper referring to this amendment and briefly explaining why this arbitrary sampling is needed.

6) Line 363: could $\rightarrow$ cloud.

We have reread the text to correct all similar typos.

7) Line 416-420: adding citations would be important to point the reader to possible mechanisms.

We have added citations to Refs. [27-30] who were cited already in Introduction.

8) Line 438: The sentence is misleading as data is not available in the github repository. A link to Google n-gram should be added as well.

We have changed the sentence, now we refer to both Google Ngrams database and our Github repository.

Yours Sincerely,

Dániel Czégel
