# Supplementary material for: Anomalous diffusion analysis of semantic evolution in major Indo-European languages
Source: PLoS One. 2024 Mar 26;19(3):e0298650. doi: 10.1371/journal.pone.0298650 (PMC10965085; doi:10.1371/journal.pone.0298650)
Supplement: Attachment — Submitted filename: response_letter_r2.pdf [file pone.0298650.s003.pdf]

Beyond Center for Fundamental Concepts in Science,
Arizona State University

Dániel Czégel
Beyond Center for Fundamental Concepts in Science
Arizona State University
Tempe, AZ 85287, USA

January 22, 2024

Haroldo V. Ribeiro
Academic Editor, PLOS ONE

Dear Haroldo V. Ribeiro,

thank you very much for your e-mail message of 4th of December 2023, related to the review of our manuscript with Submission ID PONE-D-23-19326R1 entitled "*Subdiffusive semantic evolution in major Indo-European languages*" by Bogdán Asztalos, Gergely Palla and Dániel Czégel, and we appreciate your decision to invite us to submit a revision of the manuscript, even though one of the reviewers recommended rejection.

Reviewer 2 raised some important questions which revealed that our argument had logical gaps that we should explain in more detail, especially regarding limitations of our methodology and the clarity of the null model. Consequently, the main changes in our resubmitted manuscript are the following:

1. We changed the title to "Anomalous diffusion analysis of semantic evolution in major Indo-European languages" to emphasize the methodology employed rather than the specific outcomes obtained.
2. We have added a new paragraph before the end of Discussion dedicated to the summary of these limitations and removed overly direct statements about semantic evolution being subdiffusive.
3. We have expanded the explanation of the purpose and results of shuffled time labels in section S6.

We thank all Referees for their outstanding work and the very valuable comments, making the revised version of the paper significantly better, which we hope is now suitable for publication. Our point-by-point response to the editorial board comments and the remarks by the Referees (reproduced in italics) are given below.

Beyond Center for Fundamental Concepts in Science,
Arizona State University

Response to the comment by the Editorial Board Member:

The same two previous reviewers have now reviewed the revised version of your manuscript. You will see that one of them has recommended the acceptance of your manuscript despite your avoidance of tracking the ergodicity problem. The other reviewer has, however, recommended rejection. In my opinion, his/her main argument is related to the comparison with the null models, especially regarding the results of SFig. 6, where your results indicate the existence of subdiffusion even after shuffling all time labels of the texts. Indeed, your results indicate an even more subdiffusive regime after shuffling the time label. This is a critical point for your study that needs to be seriously considered and better clarified to proceed further with the publication of your manuscript. It is not clear to me how you can possibly address this issue, but given your discussion in comparison with the usual diffusion, I am sure you also believe that this is a substantial limitation of your findings.

We sincerely appreciate the detailed feedback in improving the manuscript. We have carefully considered your suggestions and have made significant revisions to the manuscript. As detailed in our response Reviewer 2, we consider diffusion as the theoretical null-model in our work, and our main claim is that semantic change deviates from it. Nevertheless, due to methodological reasons, a measurable lower bound of $\langle\alpha\rangle$ also has to be investigated, and this is what we do in section S6. Hence, the results of Fig S6 and Fig S8 does not indicate a "stronger subdiffusion", but a baseline marked out by temporally uncorrelated time series. In order to make this argument clear, we have expanded the text of the manuscript, and section S6 in the Supporting information.

In sum, whenever semantic embedding methods are used in a diachronic context, alignment of embeddings is necessary. This introduces a similarity bias between subsequent timesteps that makes even totally independently sampled data dependent. In terms of the anomalous diffusion exponent, it introduces a non-zero "baseline" anomalous diffusion exponent that sets the lower bound for any semantically embedded and aligned time series. We observe a significant deviation from that baseline in the non-randomized (real) semantic trajectories in mean anomalous diffusion exponents as well as in the fluctuations of the trajectories.

We agree with Reviewer 2 and the Editor that our conclusions were too strong. We changed the title to "Anomalous diffusion analysis of seman-

tic evolution in major Indo-European languages". We also removed overly direct statements about semantic evolution being subdiffusive whenever it appeared without further explanation/context.

We also added a paragraph about limitations of this study, as we believe that the pipeline and its limitations are at least as informative of future work as the subdiffusive result itself.

Response to Reviewer 1:

Although the authors did not accept the challenge of dealing with ergodic and memory mechanisms in semantic subdiffusive evolution, the paper has increased its overall quality after the revision. Based on the comments of the first revision and the author's replies, the recommendation is to accept. In summary, the work applies the concept of anomalous diffusion to social systems, showing the versatility of the tools of statistical mechanics. However, if the mechanisms of subdiffusion had been approached, the relevance of the work would be even higher.

Thank you for the feedback and the suggestions given in the previous revision. We believe these significantly helped to present our narrative in a scientifically more relevant way.

Response to Reviewer 2:

This manuscript claims that the process of evolution of the meaning of words is subdiffusive. The data analysis shows that (i) the characterization of subdiffusion is very limited and unreliable; and (ii) other effects not related to the semantic evolution in time lead essentially to the same observation (e.g., stop words or randomized temporal order lead to the same type of subdiffusion). Therefore, even if the manuscript contains detailed and through data analysis, I do not recommend publication in PLOS ONE because the claims are not supported by the analysis.

Thank you for the thorough comments. We can see that a part of the claims in the previous version of our manuscripts did not adjust to the limitations of the characterization of subdiffusion and that our explanation needed some more details in a few places to be clear. Consequently, we have expanded the text with some more detailed explanation, and changed the title of the manuscript, to give our results a more accurate narrative.

(i) The signature of anomalous diffusion is a non-linear growth of the mean-squared displacement over a variety of time scales. This manuscript esti-

mates subdiffusion in only one order of magnitude and finds that that value of the exponent depends on the window size (against expectation from simple subdiffusive processes). Their choice for stopping the analysis at the year 1950 is not consistent. First, they argue that they can't go to larger time scales because they need around 10,000 words for the analysis, but at the same time the results in Fig. 1 show that the main observations are visible even for single words. Moreover, the choice of 10,000 words is justified by the vocabulary of a native speaker, a connection that seems irrelevant to this context.

We acknowledge that our results have their own limitations, so to facilitate comprehension for the reader, we have added a new paragraph before the end of Discussion dedicated to the summary of these limitations. (Specifically, we have highlighted three primary factors: the unavoidable bias of the alignment, the lack of interpretability of individual word trajectories, and the length of the analyzed time period.) Also, we have rephrased some sentences at different points of the manuscripts which had suggested stronger conclusions than these limitations allow us to draw. Furthermore, we have changed the title of the manuscript to emphasize the methodology employed rather than the specific outcomes obtained.

The window size dependence of the anomalous diffusion exponent looks unusual, but it can be attributed to the fact that we apply temporal grouping on data (panels d and e in Fig 2) before the embedding step (panel g in Fig 2), which is a non-linear operation. More specifically, word embedding is not even a data processing operation but the conversion of abstract linguistic information to a quantitative representation. This inevitably causes the window size to have a hard-to-investigate effect on the value of ¡alpha¿, but as we point out, subdiffusive behavior remains, indicating that conditions i)-iv) are violated on a fundamental, linguistic level. Quantitative comparison with simple subdiffusion models do not tell us much about our results, because these models (e.g. fractional Brownian motion or continuous-time random walk) are defined directly in geometric spaces, so such conversion is not needed, and simple linear temporal grouping would not affect the exponent in their case. (This is why we did not even detail it in our manuscript).

Our argument about the choice of 10,000 words in the previous version was a bit vague indeed, so we have rewritten the concerning paragraph in Temporal grouping in Methods. The objective was to ensure that the embedding model learns at least about as many words as a native speaker knows, because the constructed vector representation of an individual word

makes sense only compared to the others. The observable results of single words shown in Fig. 1 are also interpretable only with a static background of other words. Also, Word2vec is typically used with vocabularies of at least tens of thousands of words (as we point out in line 38), and so its working process and hyperparameters are optimized for this amount of data.

(ii) The authors find subdiffusion in stop words (Figure S2), a case in which the "semantic evolution" is unclear or expected to behave differently. Subdiffusion is also found after shuffling all time labels of the texts (Figure S6). The authors then argue that the value of the exponent is different from their observations and that therefore their conclusions can remain unchanged. However, the significance of the exact value of the exponent is not relevant to their argument (and it is not reliable, as argue in point i above). More importantly, the conclusion about the subdiffusive nature of the semantic evolution would apply equally well for smaller alpha (in fact, it is even more sudiffusive?). In the abstract, the authors make a contrast to diffusing particles, but it is clear from their analysis that a normal diffusion is not what is observed in many null-models.

Stopwords are usually filtered out before NLP investigations because frequent grammatical words with no semantic role would bias the statistics of words with real meaning. We argue in section S3 in Supporting Information that subdiffusion remains in the presence of stopwords, because they adapt to the subdiffusing environment due to the lack of semantic meaning. However, as we mention at the end of the section, one must be careful, because this part of NLP is unclear (as the Reviewer also points out). Hence, we do not believe that stopwords not resolving subdiffusive behavior would invalidate our results.

In the case of shuffled time labels we expect no temporal correlations between consecutive positions, so we expect $\langle \alpha \rangle = 0$ which is increased by the unavoidable alignment step. This non-zero baseline is intended to be determined in Fig S6 and the newly-added Fig S8, so their "small alpha" does not mean a "stronger subdiffusion", but a lower bound for measurable alpha values. We have expanded the explanation in the first paragraph of section S6 to make this argument more explicit, also, we have added a new paragraph at the end of Results where we resume this comparison and its results.

Although the above comparison has important consequences from a technical point of view, its main importance is to validate that the observed

$\langle \alpha \rangle \approx 0.4 - 0.5$ is actually above the lower bound of measurable values. Our main claim in the manuscript however is that the analysis of semantic change reveals a significant deviation from diffusion that would be expected in case of any walking phenomenon fulfilling conditions i)-iv) detailed in the paragraph starting in line 80. Hence, diffusion can be considered as a theoretical null-model in our work, and this is why we focus on it in the paper and highlight it in the abstract.

The detailed and careful data analysis present in this manuscript is of potential interest and should be published, but the presentation and interpretation given to them in this manuscript is not consistent with the data analysis reported by the authors.

We hope the latest upgrades of the manuscript, and the above explanations have improved the presentation of the data analysis to be consistent with the claims made in the text.

Yours Sincerely,

Dániel Czégel
